# Carbon and nitrogen additions induce distinct priming effects along an organic-matter decay continuum

**DOI:** 10.1038/srep19865

**Published:** 2016-01-25

**Authors:** Na Qiao, Xingliang Xu, Yuehua Hu, Evgenia Blagodatskaya, Yongwen Liu, Douglas Schaefer, Yakov Kuzyakov

**Affiliations:** 1 Key Laboratory of Tropical Forest Ecology, Chinese Academy of Sciences, Xishuangbanna Tropical Botanical Garden, Menglun, Mengla, Yunnan 666303, China; 2 Key Laboratory of Ecosystem Network Observation and Modeling, Institute of Geographic Sciences and Natural Resources Research, Chinese Academy of Sciences, 11A Datun Road, Chaoyang District, Beijing 100101, China; 3 Department of Soil Science of Temperate Ecosystems, and Department of Agricultural Soil Science, University of Göttingen, Göttingen, Germany; 4 Key Laboratory for Earth Surface Processes, Ministry of Education, Peking University, Beijing 100871, China; 5 Graduate School of the Chinese Academy of Sciences, 19A Yuquan Road, Beijing 100049, China; 6 Institute of Physicochemical and Biological Problems in Soil Science, Russian Academy of Sciences, Institutskaya, 26 Pushchino, Moscow Region, Russia, 142290

## Abstract

Decomposition of organic matter (OM) in soil, affecting carbon (C) cycling and climate feedbacks, depends on microbial activities driven by C and nitrogen (N) availability. However, it remains unknown how decomposition of various OMs vary across global supplies and ratios of C and N inputs. We examined OM decomposition by incubating four types of OM (leaf litter, wood, organic matter from organic and mineral horizons) from a decay continuum in a subtropical forest at Ailao Mountain, China with labile C and N additions. Decomposition of wood with high C:N decreased for 3.9 to 29% with these additions, while leaf decomposition was accelerated only within a narrow C:N range of added C and N. Decomposition of OM from organic horizon was accelerated by high C:N and suppressed by low C:N, but mineral soil was almost entirely controlled by high C:N. These divergent responses to C and N inputs show that mechanisms for priming (i.e. acceleration or retardation of OM decomposition by labile inputs) vary along this decay continuum. We conclude that besides C:N ratios of OM, those of labile inputs control the OM decay in the litter horizons, while energy (labile C) regulates decomposition in mineral soil. This suggests that OM decomposition can be predicted from its intrinsic C:N ratios and those of labile inputs.

Decomposition of organic matter (OM) derived from plant residues at various stages of degradation strongly contributes to structure and function of terrestrial ecosystems, providing energy and carbon (C) for microbial functioning and recycling essential nutrients for plants and microorganisms^1^. Because the terrestrial C pool is much larger than that in the atmosphere, accelerated decomposition of OM would increase atmospheric CO_2_ concentrations and lead to positive climate feedbacks^2^. This emphasizes the need for better understanding of factors affecting OM decomposition.

Microbial demands for C, N and P have been thought to follow narrow ratios. This is reflected by microbial stoichiometry^3^,^4^, which overrides microorganisms as a regulator of soil C and N cycling^5^. Microbial decomposers quickly respond to labile C and nitrogen (N) inputs from root exudates or decomposing litter, and N deposition, but soil organic C turnover shows variable responses; often accelerated by labile-C additions^6^ and slowed by added N^7^,^8^ when considered separately. In contrast, leaf litter decomposition can be slowed by labile C^9^, but generally accelerated by N additions^10^. Nitrogen effects on wood decomposition are mostly negative^11^, but labile C there has not been examined.

Such phenomena (*i.e*. OM decomposition is modified by labile inputs) are described as priming effects^12^. Priming can greatly affect global C storage^13^ through modifying soil OM decomposition, especially shown by rhizosphere priming^14^,^15^. However, most previous priming studies examined soil OM after separate additions of labile N^16^ or C^17^. However, labile C and N are always both present at different amounts and ratios in terrestrial ecosystems. Thus it is necessary to clarify how priming responds to amounts and ratios of labile resource inputs for a better understanding of OM decomposition. Some studies have considered labile C and N together, but they examined only narrow C:N ratios corresponding to microbial biomass for priming of SOM decomposition^18^,^19^,^20^. As microbial decomposers use C for both energy and anabolism^21^,^22^,^23^, higher decomposition rates should occur at considerably higher C:N ratios in labile inputs^24^. However, this has not been explored in earlier studies.

Additionally, amounts and ratios of labile C and N inputs vary globally over a wide range. Labile C inputs are controlled by local net primary productivity^25^ through decomposing litter and root exudates, while labile N inputs primarily result from N_2_ fixation by legumes, fertilization and N deposition at regional scales^26^. The full range of C:N ratios in labile inputs may span from 0.4 to 200, but a global map remains to be developed (see Supplementary materials).

Besides labile inputs, organic matter in soils is a highly heterogeneous mix of detritus from plants, including living organisms with the products of their metabolic activities^1^. In terrestrial ecosystems, plant litter often deposits on the organic horizon with mineral soil beneath (Fig. 1). Their continuous decomposition and transformations develop a decay continuum^27^. Along this continuum, decomposing OM is at various stages of degradation and has distinct properties. Plant litter has high C:N ratios; woody materials range from less than 100 to more than 500 (ref. 28), and leaves from 10 to 100 (ref. 29). Those of soil OM are much narrower and centered approximately on 17 (ref. 4). Although C:N ratios of various OM substrates range widely, those of microbial decomposers are much narrower (centered on 7:1, ref. 4). Several studies have suggested that microorganisms cope with this C:N imbalance between OMs and their biomass through regulating their C- and N-use efficiencies^30^,^31^. However, it remains unknown how microbial decomposition of heterogeneous OMs with wide C:N ratios responds to varying ratios of labile C and N inputs along OM decay continuum.

Studying decomposition of all OMs in response to the full global range of labile C and N inputs can substantially improve our knowledge of OM turnover and their consequences. Therefore, we measured decomposition rates of wood, leaf litter, and OM in both organic and mineral soil horizons along the decay continuum (Fig. 1), adding labile C and N inputs over potential global ranges of C:N ratios. Decomposition was assessed as cumulative CO_2_ production, with isotopically labeled C to distinguish OM substrate C from labile-C inputs. We aim to clarify: (1) how OM decomposition responds to labile C and N inputs, and (2) what controls OM decomposition along the decay continuum. Furthermore, we demonstrate priming stoichiometry in three dimensions (C:N ratios in OM, C:N ratios in labile inputs, and priming intensity) for a better understanding OM decomposition.

## Results

Four heterogeneous OM substrates collected from a decay continuum of a subtropical forest in China had widely differing C:N ratios (Table S1) and decay rates (Fig. 2 inset). Decomposition rates (water-only additions) of these four OMs during laboratory incubations ranged from 0.8% in mineral soil to 4.3% in leaf per month (Fig. 2 inset). This suggests decreased decomposability of these heterogeneous OM forms along the decay continuum.

Decomposition of all four OMs responded to labile C (glucose) and N (ammonium) inputs in distinct ways (Fig. S1). All additions of C and N slowed freshly cut wood decomposition, but the lowest and highest additions of both C and N caused smaller negative priming (Figs 2 and 3a, Tables S2 and S3). Leaves showed the largest priming with smallest additions of both C and N, while higher labile inputs caused less priming (ranging from -6 to 8% compared to water-only additions). Priming was positive only within a narrow range of C:N ratios in labile inputs to leaves (Figs 2 and 3b). Decomposition of both these plant litters responded more strongly to C:N ratios in labile inputs than did soils (Fig. 4). Surface organic soil showed slightly negative priming (−4%) with labile inputs at the lowest C:N ratio, and very large positive priming at the highest C:N ratio of 320 (134%) (Figs 2 and 3c). Priming in 0 to 10 cm mineral soil showed little response to C:N ratios in the labile inputs, but responded most to labile C additions (Figs 3d,4), to the maximum of 83% (Fig. 2). This reflects that mineral soil was less affected by labile N inputs than were any other components of this OM continuum (Fig. 4). Overall, proceeding from leaf to wood, organic soil, and mineral soil, the contribution of labile C inputs to priming substantially increased, while that of labile N inputs and C:N ratios decreased (Fig. 4).

With all results considered together, clear and novel priming patterns appear for C:N ratios in both labile inputs and decomposing organic matter (Fig. 5). With low labile C:N input ratios (less than 55), priming had relatively minor differences among the four OM forms (Fig. 5; horizontal black line). Conversely, with high labile C:N input ratios, priming was strongly negative for the OM with high C:N ratios and strongly positive for those with lower C:N ratios (Fig. 5; horizontal white line). Priming effects were negatively correlated with intrinsic C:N ratios in decomposing organic matter. The threshold of C:N ratio in OM substrates where priming changed from positive to negative here was about 55 (Fig. 5).

## Discussion

### Patterns within heterogeneous organic forms

We explored effects of labile C and N inputs on decomposition of plant litter and soil OM along the decay continuum over a wide range of C:N ratios in labile inputs. To our knowledge, this is the first analysis of decomposition stoichiometry of heterogeneous OM along a decay continuum in three dimensions (C:N ratio in organic matter, C:N ratio in labile inputs, and priming intensity) (Fig. 5).

We found that mechanisms previously invoked to explain priming (*e.g.* preferential substrate utilization^12^,^32^, microbial community shifts^33^, mining of N^34^, C starvation^23^, and microbial activation^18^) play different roles along the OM decay continuum. Negative priming of wood-litter decomposition could be ascribed to preferential substrate utilization and microbial community shifts^32^,^33^,^35^,^36^. Wood contains a substantial amount of lignin, and thus is less microbially available than leaf litter^28^. With labile C and N both added to wood, microbial communities might switch from lignin to more readily available components and slow decomposition. Priming of leaf-litter decomposition is reduced by high labile C input under low N input condition and also by high N input under low C input condition (Fig. S1). This suggests that preferential substrate utilization and mining of N are both involved in priming of leaf-litter decomposition (Fig. 3b). A previous study showed that individual additions of C, N, or P did not accelerate leaf decomposition, but all combined additions did^37^, further illustrating that C:N ratios in the labile inputs are important for plant-litter decomposition (Figs 4 and 5).

Priming in organic soil was strongly enhanced by high C:N labile inputs but apparently slowed by low C:N labile inputs. This indicates that high C inputs lead to microbial N mining^34^, probably through distinct N-mining responses of soil microorganisms depending on C:N ratios in the substrate^38^. This process can be typically reduced by high labile N additions, *e.g.* under elevated CO_2_ (elevated root exudation), N addition reduced rhizosphere priming^39^. Additionally, priming caused by higher additions of labile C was reduced with N additions to agricultural soils^40^. Priming in our organic soil horizon was highest with high C:N labile inputs. This supports recent conclusions that rapid soil OM decomposition under elevated CO_2_ is caused by increased root exudates rich in labile C^41^.

A previous study showed that adding root-mimic resources at C:N of 10 to mineral soil of a temperate forest induced stronger priming than C-only addition^42^. However, in this study mineral-soil OM decomposition responded mostly to labile C inputs, where energy deficiency^23^ (*i.e*. low C availability) strongly controlled microbial decomposition in our subtropical forest (Figs 3d and 4). The discrepancy might relate to microbial C depletion at higher temperature in the subtropical forest. Carbon depletion by microbial processing substantially decreases intrinsic C:N ratios in OMs along the decay continuum^4^. The C:N ratio in our organic soil horizon was only slightly higher than in mineral soil horizon (19 vs. 16), but distinct priming mechanisms appear to be responsible in organic and mineral soils. Clay minerals are abundant in mineral soils but absent from the surficial organic layer. OM substrates occluded within clay minerals can be protected from microbial decomposition^13^,^43^, leading to different priming mechanisms.

### Priming patterns along a decay continuum

**Figure 1:**
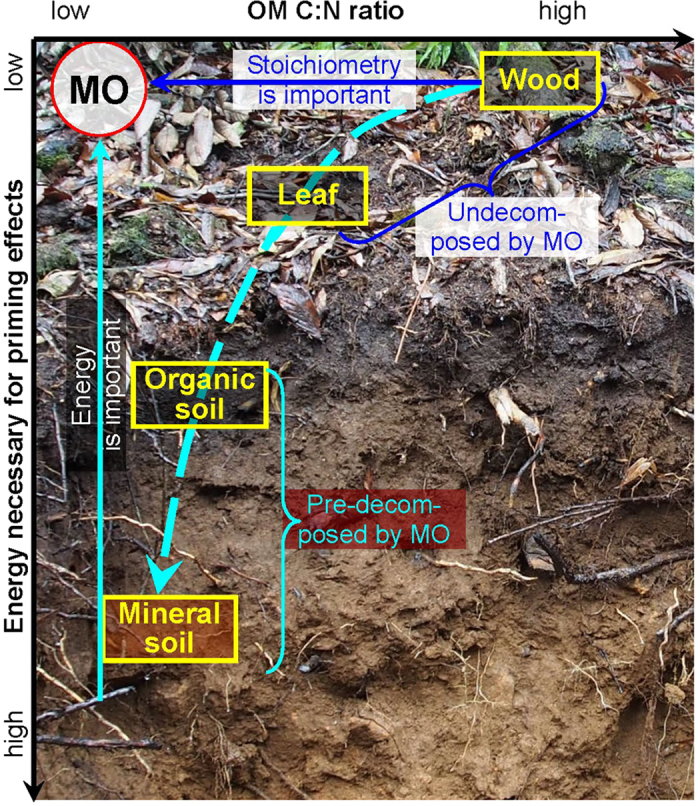
Conceptual model of organic-matter decomposition priming. Cyan arrow indicates energetic limitations to decomposing microorganisms (MO) for priming of organic matter (OM) decomposition. Blue arrow indicates decreasing stoichiometric limitations to microorganisms for priming of OM decomposition. Photograph was made by Chuansheng Wu with his permission to use it.

Distinct mechanisms responsible for priming of heterogeneous OM decomposition suggest that microbes decomposing them respond differently to labile C:N supplies and ratios^44^. Such responses depend on whether OM has been previously decomposed by microorganisms^31^. Fresh leaf and wood litter have not previously undergone microbial transformations. They are rich in C and energy, with wood containing much less N. With increased microbial processing along the decay continuum, microbial decomposers in soil (Fig. 1) become C and energy limited^23^. Therefore, the importance of labile C for priming substantially increases, while that of N and C:N ratios in the inputs decreases along the decay continuum with increased microbial processing (Fig. 4).

**Figure 2:**
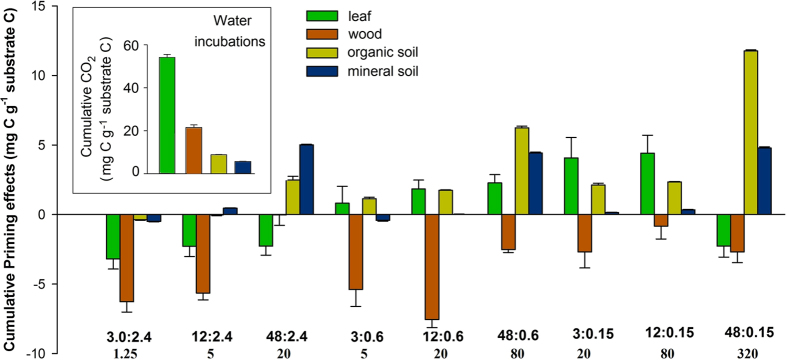
Priming by labile carbon and nitrogen inputs to leaf, wood, and organic and mineral soils. Labile carbon and nitrogen inputs are presented as mg g^−1^ substrate C, with ratios below. Inset shows decomposition of these substrates incubated with water-only controls. Detailed statistical information was presented in Tables S2 and S3 in the Supplemental materials.

**Figure 3:**
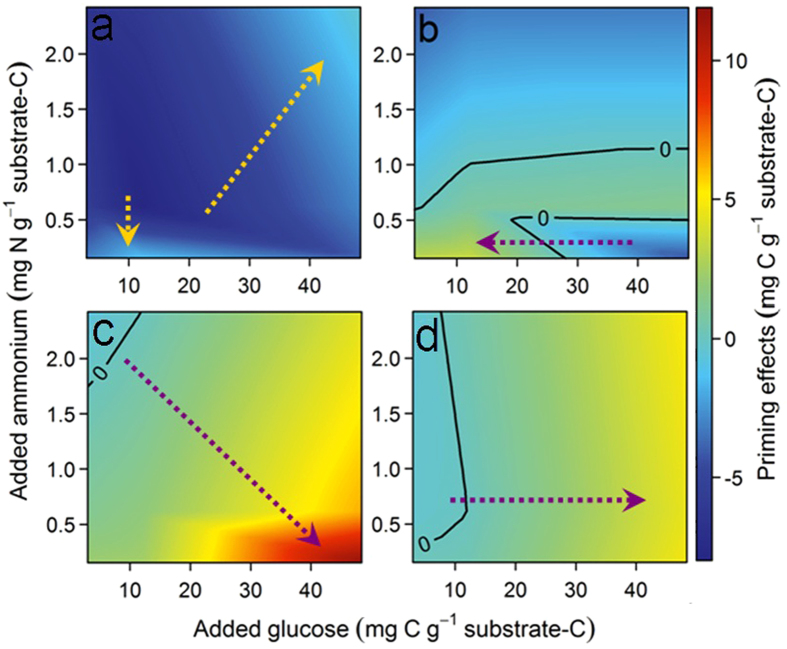
Priming patterns resulting from different glucose and ammonium inputs to incubations of wood litter (a), leaf litter (b), as well as Oa horizon organic soil (c) and mineral-soil A horizon (d) from a subtropical forest. Zero lines indicate where CO_2_ release did not differ from the water-only control incubations. Dotted-line arrows show gradients with largest priming effects from these carbon and nitrogen additions. This figure was made using the data from nine treatments for each OM form in Fig. 2 to create a ‘point’ (color) in its panel, with the rest of each panel interpolated from those 9 points.

**Figure 4:**
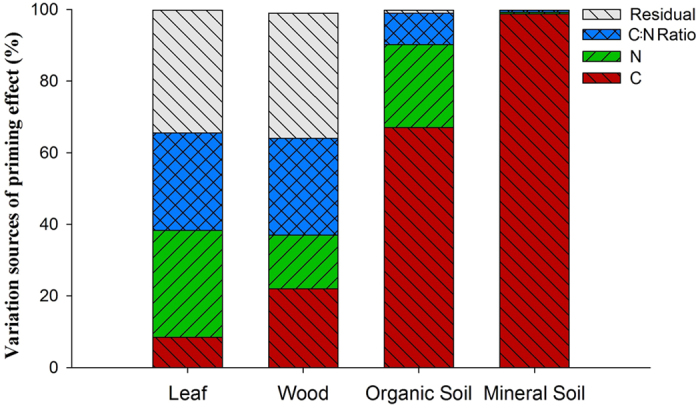
Contributions of glucose (C), ammonium (N), their interactions, and unexplained residual variation to priming effects in each of the four heterogeneous organic substrates (leaf litter, wood litter, and organic and mineral soils).

**Figure 5:**
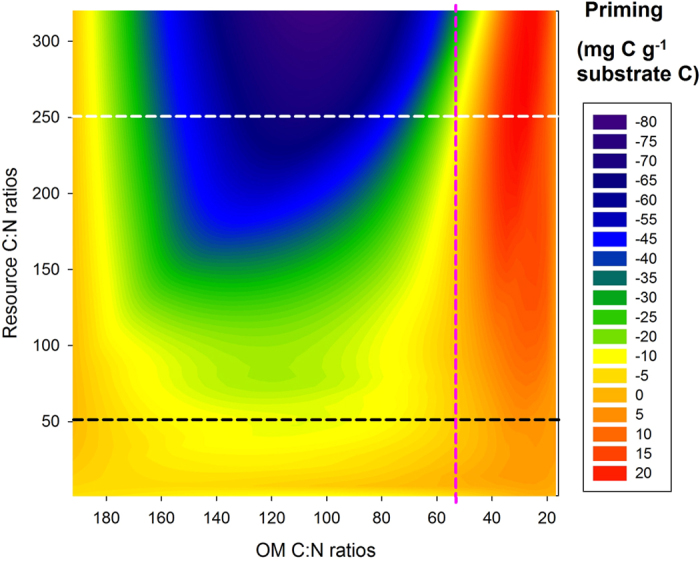
Responses of priming of organic matter (OM) decomposition to OM C:N ratios (horizontal axis) and labile C:N ratios (vertical axis). This contour figure was made based on all priming results of four OM forms from Fig. 2, using C:N ratios in OMs as x-axis, C:N ratios in the labile inputs as y-axis, and all priming data as z (color) axis. Priming effects vary strongly among substrates along the white dashed line, where labile carbon inputs are high and nitrogen is low. Priming effects do not vary strongly among substrates along the black dashed line where labile carbon is low and nitrogen is high. The dashed pink line indicates the substrate C:N threshold ratio where priming changes from negative to positive.

A recent study showed that microbial C:N ratios and microbial demands for C and nutrients modify the magnitude and direction of priming^45^. Imbalances between microbial C:N and those in decomposing OMs appear to regulate decomposition rates^4^. However, this regulation can be of minor importance under natural conditions where microorganisms demonstrate broad stoichiometric flexibility and the potential for community shifts. Furthermore, we find differences among decomposing OMs to be highly important (Fig. 5). Consequently, ratios of C:N in OMs and labile inputs clearly reflect the direction and intensity of priming (Fig. 5). This leads to a new hypothesis: microbial decomposition of OMs first relies on whether they have been microbially processed before (Fig. 2 inset), and then depends on the interactions between C:N ratios in OM forms and those in labile inputs (Fig. 5). Owing to complicated interactions between microorganisms, OMs and labile inputs, no single mechanism can explain the different patterns of priming of all forms of OM. The mechanisms appear to depend strongly on intrinsic properties of heterogeneous OM. We conclude that decomposition of previously undecomposed OM (*i.e.* plant litter) is regulated by C:N stoichiometry in the labile inputs, while that of the most decomposed OM (*i.e.* mineral soil) is controlled by energy (Figs 1,3 and 4).

For labile C:N ratios less than 55, we found small differences in priming along the OM decay continuum (Fig. 5). Priming changed from negative to positive with substrate C:N ratios also below about 55 (Fig. 5). Whether this indicates a fundamental property of microbial decomposition in response to labile inputs needs to be determined in future studies.

### Global patterns of OM decomposition

We observed priming effects differing most strongly across the OM continuum with high labile C and low N inputs (high C:N ratios), and they were consistently smaller with low labile-C supplies and high N (this is the input threshold; Fig. 5). Labile C and N inputs vary widely across the globe (Supplementary materials) and we suggest that decomposition may be accelerated in areas with high C and low N inputs (tropical ecosystems far from anthropogenic inputs). Smaller decomposition changes may occur in regions receiving low C and high N (high-latitude agricultural ecosystems). We propose that OM priming can be predicted in terrestrial ecosystems on the basis of C:N ratios of both labile inputs and decomposing OM. Priming of OM decomposition is not geographically uniform^13^, and can vary further with future global changes in plant productivity and N deposition.

Our study provides a basis for incorporating stoichiometric responses of microbial decomposition and energy demand into conceptual and mathematical models. Such energy and stoichiometry based models should consider future changes in available C and N supplies to better predict OM dynamics, C sequestration, and potential C-climate feedbacks.

## Methods

Detailed methods are presented in the supplementary materials

### Collecting organic substrates

Four organic substrates (*i.e.* organic soil horizon, mineral soil horizon, leaf litter, and wood litter; Table S1) were collected from a subtropical broad-leaved evergreen forest in the Ailao Mountains Nature Reserve (24°32′N, 101°01′E), 2476 m above sea level, Yunnan Province, in southwestern China. This site is characterised by a monsoon climate, with distinct cool/dry seasons from November to the following April and warm/wet seasons from May to the following October. Over the past 20 years, the annual average precipitation was 1840 mm, and the annual mean air temperature was 11.3 °C. Soils are Alfisols with properties described in Table S1. This forest is dominated by *Lithocarpus chintungensis*, *Rhododendron leptothrium*, *Vaccinium duclouxii*, *Lithocarpus xylocarpus*, *Castanopsis wattii*, and *Schima noronhae*. Organic soil was collected from O_a_ horizon (*ca*. 7-cm thickness), whereas mineral soil was collected from the upper 10 cm of the A horizon. Plant litter was collected from the O_i_ horizon (the litter floor). Undecomposed mixed leaf litter was collected, and wood litter was a mixture of three locally dominant tree species (*Lithocarpus chintungensis, Lithocarpus xylocarpus*, and *Schima noronhae*).

### Experimental design

Labile C as glucose was added to these organic substrates at three levels (*i.e*. 0.3, 1.2, and 4.8% of their individual organic-C contents). Available N was also added at three levels (*i.e*. 0.15, 0.6, and 2.4% of their individual organic-C contents). NH_4_Cl was added because ammonium dominates inorganic N in this soil (Table S1), and it is the preferred N source for bacteria and fungi. As a result, three levels of C and N additions produced nine combinations in total for each substrate, with a control treatment involving the addition of only water (Table S4). Preliminary experiments with these substrates showed few significant phosphorus (P) effects; therefore, P was added at N:P = 10:1 throughout to avoid P limitation^46^.

### Incubation

Soils collected from organic and mineral horizons were separately sieved (2 mm), and visible plant materials were removed manually; soils were then thoroughly homogenised. Leaf litter was air dried and cut to *ca*. 1-cm pieces, and wood litter was reduced to *ca.* 0.5 × 3 cm for introduction into incubation bottles. Initial chemistry of these incubated materials is described in supplemental Table S1. Each substrate was incubated in 330-mL bottles in the laboratory at 23 ± 1 °C with labile C, N, and P dissolved, or with an equal amount of water (control). To allow for the complete trapping of CO_2_ for mass spectrometric analysis, we used 2.5 g leaf litter and 3 g wood and 30 g organic or mineral soils, based on results from preliminary experiments. Labile C was added initially as uniformly labelled ^13^C-glucose; N, as NH_4_Cl; and P, as Na_2_HPO_4_. Incubations of leaf and wood were each inoculated with 0.2 g organic soil to ensure the presence of natural microbial decomposer communities.

### CO_2_ efflux measurements

CO_2_ effluxes released from these incubated OM materials were measured using LI820 IRGA (LiCor, Lincoln, Nebraska, USA). At early stages of incubations, they were measured more frequently while they were done with longer intervals at late stages. Incubation measurements were ended when CO_2_ production in resource-addition incubations no longer differed from the corresponding controls^47^. Total incubation times were 528 h for mineral soils, 676 h for organic soils, 720 h for wood litter, and 915 h for leaf litter. Detailed timing of CO_2_ efflux measurements for each OM form was presented in Table S5. There were 6 to 8 replicates for each treatment. During the incubations, CO_2_ was repeatedly trapped in NaOH for ^13^C measurements with three or more replicates.

### Carbon isotope analysis

The C-isotope ratios of trapped CO_2_ in NaOH solutions were measured after SrCl_2_ precipitation, followed by followed by isotope ratio mass spectrometry (MAT253, Finnigan MAT, Bremen, Germany).

### Calculations and statistics

A mixing model was used to calculate the fractions of CO_2_-C derived from OM substrates and from added glucose (C_glucose_)^48^. This model allows the variability from isotope measurements to be combined with that from CO_2_ flux measurements. Primed C was calculated as follows:


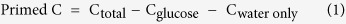


where C_total_ is total C-CO_2_ from glucose-treated OM substrates, C_glucose_ is C-CO_2_ derived from added glucose, and C_water only_ is total C-CO_2_ from the OM substrates receiving only water^49^.

Shapiro–Wilkinson tests confirmed that all data distributions were normally distributed. Priming differences among substrates under the same treatment (Table S2), as well as those among C and N addition levels within a single substrate (Table S3), were detected by *post-hoc* Tukey HSD tests (P < 0.05). The effects of labile C and N additions on cumulative priming of four OM substrates were investigated by two-way analysis of variance (ANOVA; Tables S6–S7). The contribution of labile C, N and their interactions to the total variance for priming was calculated by dividing the respective type III sum of squares by the total sum of type III sum of squares from two-way ANOVA results from Table S6.

## Additional Information

**How to cite this article**: Qiao, N. *et al.* Carbon and nitrogen additions induce distinct priming effects along an organic-matter decay continuum. *Sci. Rep.* **6**, 19865; doi: 10.1038/srep19865 (2016).

## Supplementary Material

Supplementary Information

## References

[b1] Swift M. J., Heal O. W. & Anderson J. M. Decomposition in Terrestrial Ecosystems (Blackwell, 1979).

[b2] Melillo J. M. *et al.* Soil warming, carbon–nitrogen interactions, and forest carbon budgets. Proc. Natl. Acad. Sci. USA 108, 9508–9512 (2011). 21606374 10.1073/pnas.1018189108 PMC3111267

[b3] Cleveland C. C. & Liptzin D. C:N:P stoichiometry in soil: is there a “Redfield ratio” for the microbial biomass? Biogeochemistry 85, 235–252 (2007).

[b4] Mooshammer M., Wanek W., Zechmeister-Boltenstern S. & Richter A. Stoichiometric imbalances between terrestrial decomposer communities and their resources: mechanisms and implications of microbial adaptations to their resources. Front. Microbiol. 5, 10.3389/fmicb.2014.00022 (2014). PMC3910245 24550895

[b5] Buchkowski R. W., Schmitz O. J. & Bradford M. A. Microbial stoichiometry overrides biomass as a regulator of soil carbon and nitrogen cycling. Ecology 96 **(4)**, 1139–1149 (2015). 26230033 10.1890/14-1327.1

[b6] Fontaine S. *et al.* Stability of organic carbon in deep soil layers controlled by fresh carbon supply. Nature 450, 277–281 (2007). 17994095 10.1038/nature06275

[b7] Sutton M. A. *et al.* Uncertainties in the relationship between atmospheric nitrogen deposition and forest carbon sequestration. Glob. Change Biol. 14, 2057–2063 (2008).

[b8] Janssens I. A. *et al.* Reduction of forest soil respiration in response to nitrogen deposition. Nat Geosci 3, 315–322 (2010).

[b9] Chigineva N. I., Aleksandrova A. V. & Tiunov A. V. The addition of labile carbon alters litter fungal communities and decreases litter decomposition rates. Appl. Soil Ecol. 42, 264–270 (2009).

[b10] Knorr M., Frey S. D. & Curtis P. S. Nitrogen additions and litter decomposition: a meta-analysis. Ecology 86, 3252–3257 (2005).

[b11] Fog K. The effect of added nitrogen on the rate of decomposition of organic matter. Biol. Rev. 63, 433–462 (1988).

[b12] Kuzyakov Y., Friedel J. K. & Stahr K. Review of mechanisms and quantification of priming effects. Soil Biol. Biochem. 32, 1485–1498 (2000).

[b13] Sulman B. N., Phillips R. P., Oishi A. C., Shevliakova E. & Pacala S. W. Microbe-driven turnover offsets mineral-mediated storage of soil carbon under elevated CO_2_. Nat. Clim. Change 4, 1099–1102 (2014).

[b14] Cheng W. *et al.* Synthesis and modeling perspectives of rhizosphere priming. New Phytol. 201, 31–44 (2014). 23952258 10.1111/nph.12440

[b15] Finzi A. C. *et al.* Rhizosphere processes are quantitatively important components of terrestrial carbon and nutrient cycles. Glob. Change Biol. 21, 2082–2094 (2015). 10.1111/gcb.12816 25421798

[b16] Löhnis F. Nitrogen availability of green manures. Soil Sci. 22, 253–290 (1926).

[b17] Bingeman C. W., Varner J. E. & Martin W. P. The effect of addition of organic materials on the decomposition of an organic soil. Soil Sci. Soc. Am. Proc. 17, 34–38 (1953).

[b18] Blagodatskaya E. V., Blagodatsky S. A., Anderson T.-H. & Kuzyakov Y. Priming effects in Chernozem induced by glucose and N in relation to microbial growth strategies. Appl. Soil Ecol. 37, 95–105 (2007).

[b19] Manzoni S., Taylor P., Richter A., Porporato A. & Ågren G. I. Environmental and stoichiometric controls on microbial carbon-use efficiency in soils. New Phytol. 196, 79–91 (2012). 22924405 10.1111/j.1469-8137.2012.04225.x

[b20] Chowdhury S., Farrell M. & Bolan N. Priming of soil organic carbon by malic acid addition is differentially affected by nutrient availability. Soil Biol. Biochem. 77, 158–169 (2014).

[b21] Sardans J., Rivas-Ubach A. & Peñuelas J. The elemental stoichiometry of aquatic and terrestrial ecosystems and its relationships with organismic lifestyle and ecosystem structure and function: a review and perspectives. Biogeochemistry 111, 1–39 (2011).

[b22] Wang G. & Post W. M. A theoretical reassessment of microbial maintenance and implications for microbial ecology modeling. FEMS Microbiol. Ecol. 81, 610–617 (2012). 22500928 10.1111/j.1574-6941.2012.01389.x

[b23] Hobbie J. E. & Hobbie E. A. Microbes in nature are limited by carbon and energy: the starving-survival lifestyle in soil and consequences for estimating microbial rates. Front. Microbiol. 4, 324, 10.3389/fmicb.2013.00324 (2013). 24273534 PMC3824246

[b24] Gao L., Sun M. H., Liu X. Z. & Che Y. S. Effects of carbon concentration and carbon to nitrogen ratio on the growth and sporulation of several biocontrol fungi. Mycol. Res. 111, 87–92 (2007). 17158041 10.1016/j.mycres.2006.07.019

[b25] Norby R. J. & Zak D. R. Ecological lessons from free-air CO_2_ enrichment (FACE) experiments. Ann. Rev. Ecol. Evol. Syst. 42, 181–203 (2007).

[b26] Galloway J. N. *et al.* Transformation of the nitrogen cycle: recent trends, questions, and potential solutions. Science 320, 889–892 (2008). 18487183 10.1126/science.1136674

[b27] Melillo J. M. *et al.* Carbon and nitrogen dynamics along the decay continuum: plant litter to soil organic matter. Plant Soil 115, 189–198 (1989).

[b28] Weedon J. T. *et al.* Global meta-analysis of wood decomposition rates: a role for trait variation among tree species? Ecol. Lett. 12, 45–56 (2009). 19016827 10.1111/j.1461-0248.2008.01259.x

[b29] Reich P. B. & Oleksyn J. Global patterns of plant leaf N and P in relation to temperature and latitude. Proc. Natl. Acad. Sci. USA 101, 11001–11006 (2004). 15213326 10.1073/pnas.0403588101 PMC503733

[b30] Sinsabaugh R. L. & Follstad, Shah J. J. Ecoenzymatic stoichiometry and ecological theory. Ann. Rev. Ecol. Evol. Syst. 43, 313–343 (2012).

[b31] Mooshammer M. *et al.* Adjustment of microbial nitrogen use efficiency to carbon:nitrogen imbalances regulates soil nitrogen cycling. Nat. Comm. 5, 3694, 10.1038/ncomms4694 (2014). PMC3997803 24739236

[b32] Cheng W. Rhizosphere feedbacks in elevated CO_2_. Tree Physiol. 19, 313–320 (1999). 12651574 10.1093/treephys/19.4-5.313

[b33] Fontaine S., Mariotti A. & Abbadie L. The priming effect of organic matter: a question of microbial competition? Soil Biol. Biochem. 35, 837–843 (2003).

[b34] Craine J. M., Morrow C. & Fierer N. Microbial nitrogen limitation increases decomposition. Ecology 88, 2105–2113 (2007). 17824441 10.1890/06-1847.1

[b35] Allison S. D. A trait-based approach for modelling microbial litter decomposition. Ecol. Lett. 15, 1058–1070 (2012). 22642621 10.1111/j.1461-0248.2012.01807.x

[b36] Dijkstra F. A., Carrillo Y., Pendall E. & Morgan J. A. Rhizosphere priming: a nutrient perspective. Front. Microbiol. 4, 1–8 (2013). 23908649 10.3389/fmicb.2013.00216 PMC3725428

[b37] Barantal S., Schimann H., Fromin N. & Hättenschwiler S. Nutrient and carbon limitation on decomposition in an Amazonian moist forest. Ecosystems 15, 1039–1052 (2012).

[b38] Murphy C. J., Baggs E. M., Morley N., Wall D. P. & Paterson E. Rhizosphere priming can promote mobilisation of N-rich compounds from soil organic matter. Soil Biol. Biochem. 81, 236–243 (2015).

[b39] Carrillo Y., Dijkstra F. A., Pendall E., LeCain D. & Tucker C. Plant rhizosphere influence on microbial C metabolism: the role of elevated CO_2_, N availability and root stoichiometry. Biogeochemistry 117, 229–240 (2014).

[b40] Chowdhury S., Farrell M. & Bolan N. Priming of soil organic carbon by malic acid addition is differentially affected by nutrient availability. Soil Biol. Biochem. 77, 158–169 (2014).

[b41] van Groenigen K. J., Qi X., Osenberg C. W., Luo Y. Q. & Hungate B. A. Faster decomposition under increased atmospheric CO_2_ limits soil carbon storage. Science 344, 508–509 (2014). 24762538 10.1126/science.1249534

[b42] Drake J. E., Darby B. A., Giasson M.-A., Kramer M. A., Phillips R. P. & Finzi A. C. Stoichiometry constrains microbial response to root exudation-insights from a model and a field experiment in a temperate forest. Biogeosciences 10, 821–838 (2013).

[b43] Schmidt M. W. I. *et al.* Persistence of soil organic matter as an ecosystem property. Nature 479, 49–56 (2011). 21979045 10.1038/nature10386

[b44] Chen R. R. *et al.* Soil C and N availability determine the priming effect: microbial N mining and stoichiometric decomposition theories. Glob. Change Biol. 20, 2356–2367 (2014). 10.1111/gcb.12475 24273056

[b45] Wang H. *et al.* Quality of fresh organic matter affects priming of soil organic matter and substrate utilization patterns of microbes. Sci. Rep. 5, 10102 10.1038/srep10102 (2015). 25960162 PMC4426597

[b46] Manzoni S. & Porporato A. Common hydrologic and biogeochemical controls along the soil–stream continuum. Hydrol. Proc. 25, 1355–1360 (2011).

[b47] Cleveland C. C., Townsend A. R. & Schmidt S. K. Phosphorus limitation of microbial processes in moist tropical forests: Evidence from short-term laboratory incubations and field studies. Ecosystems 5 **(7)**, 680–691 (2002).

[b48] Phillips D. L., Newsome S. D. & Gregg J. W. Combining sources in stable isotope mixing models: alternative methods. Oecologia 144, 520–527 (2005). 15711995 10.1007/s00442-004-1816-8

[b49] Qiao N. *et al.* Labile-carbon retention compensates for CO_2_ released by priming in forest soils. Glob. Change Biol. 20, 1943–1954 (2014). 10.1111/gcb.12458 24293210

