# Supplementary material for: Carbon and nitrogen additions induce distinct priming effects along an organic-matter decay continuum
Source: Sci Rep. 2016 Jan 25;6:19865. doi: 10.1038/srep19865 (PMC4726261; doi:10.1038/srep19865)
Supplement: Supplementary Information [file srep19865-s1.doc]

**Supplementary material for**

**Carbon and nitrogen additions induce distinct priming effects**

**along an organic-matter decay continuum**

Na Qiao, Xingliang Xu, Yuehua Hu, Evgenia Blagodatskaya, Yongwen Liu, Douglas Schaefer, Yakov Kuzyakov

**METHODS**

**Site description**. For this laboratory-incubation study, four organic substrates were collected from a subtropical broad-leaved evergreen forest in the Ailao Mountains Nature Reserve (24°32’N, 101°01’E), 2476 m above sea level, Yunnan Province, in southwestern China. This forest is characterized by a monsoonal climate, with distinct cool/dry (November to April) and warm/wet (May to October) seasons (Zhang 1983). Annual average precipitation was 1780 mm and annual mean air temperature was 11.3 °C over the past 20 years. Soils are Alfisols. Soils are classified as loamy Lixisols (WRB 2006) or Ustalf Alfisols by USDA soil taxonomy, with properties described in **Table S1.** They are derived from weakly metamorphosed Permian marine sediments. The forest canopy is dominated by *Lithocarpus* *chintungensis*, *Rhododendron* *leptothrium*, *Vaccinium* *ducluoxii*, *Lithocarpus* *xylocarpus*, *Castanopsis* *wattii*, and *Schima* *noronhae* (Wu 1980).

**Sample collection**. Undecomposed leaf litter was collected from the O_i_ horizon without reference to species, and wood litter was a mixture of three locally dominant tree species (*Lithocarpus chintungensis, Lithocarpus xylocarpus*, and *Schima noronhae*). Organic soil was collected from combined O_a_ and O_e_ horizons (indistinguishable in this forest soil and *ca*. 7 cm thick) and mineral soil from the top 10 cm of the A horizon. Therefore, these four heterogeneous OMs represent different stages of OM decomposition. Substrate chemistry was analysed for this project except for soil available C (Chan et al. 2006; Feng et al. 2011) and is presented in (Table S1).

**Sample preparation**. Organic and mineral soil layers were separately sieved (2 mm), visible plant materials removed manually, and each was thoroughly homogenized. Thirty grams of air-dried, 2-mm sieved, root-removed organic and mineral soils were weighed into 330 mL incubation bottles. Leaf litter was air dried and cut to *ca*. 1 cm pieces. Wood was cut to 2 cm pieces, and each ‘litter’ bottle received 0.2 g organic soil to ensure establishment of natural microbial decomposer communities. Carbon and C-isotope fluxes from the organic soil inocula to leaf and wood substrates were calculated based on incubation data of mineral soil and subtracted prior to data analysis. To avoid CO_2_ flush from sieving and other manipulations, all of these OMs were pre-incubated at 23°C for 3 days.

**Incubations with labile C and N additions.** In this study, we defined labile (C and N) resources as soluble and not requiring processing by exoenzymes before microbial uptake. OM substrates are C- and N-containing plant-derived organic polymers that must undergo exoenzymatic processing before microbial uptake (Burns et al. 2013). Thus, only substrates can undergo priming. These four heterogeneous OMs were incubated in the laboratory. Each substrate was incubated in 330-mL bottles in the laboratory at 23±1 ºC with dissolved C, N, and P or with only water (control). Based on preliminary experiments, we used 2.5 g leaf litter, 3.0 g wood and 30 g organic or mineral soils to allow for complete trapping of CO­_2_ for mass spectrometric analysis. Labile C was added to these OMs at low, intermediate and high levels (*i.e*. 0.3, 1.2 and 4.8% of their individual organic-C contents). Labile C was uniformly-labeled ^13^C-glucose (δ^13^C = 944‰ to mineral soil and 100‰ to other substrates). At each of these C-addition levels, N and P were also added at three levels, constituting 9 treatments in total and one water-only control (Table S4). Available N was added as NH_4_Cl because it dominates inorganic N in this soil. Phosphorus was added as Na_2_HPO_4_. Preliminary incubations with these four substrates showed few significant differences among N:P ratios, therefore an intermediate value of 10 (Manzoni and Porporato 2011) was used throughout. More extreme N:P ratios are thought to favor either fast-or slow-growing microbes (Güsewell and Gessner 2009). There were only initial resource additions. There were 6 to 8 replicates in each treatment. Therefore, this study was a three-factorial experiment based on OM types, C and N amounts and ratios, with N:P ratio held constant. Incubation of each of the four heterogeneous OMs was continued until CO_2_ from resource additions no longer differed significantly from their corresponding control treatments (Cleveland et al. 2002). Those total times were 529, 676, 720 and 915 hours for organic soil, mineral soil, wood, and leaves, respectively (Table. S5), increasing in the same order as decomposition in the control treatments (Fig. 2 inset).

**CO_2_ efflux measurements.** CO_2_ effluxes released from these incubated OM materials were measured using LI820 IRGA (LiCor, Lincoln, Nebraska, USA). At early stages of incubations, they were measured more frequently while they were done with longer intervals at late stages. Detailed timing of CO_2_ efflux measurements for each OM form was presented in Table S5. At frequent intervals during the incubations, incubation bottles were briefly capped and internal CO_2_ accumulation rates were measured. Linear CO_2_ concentration increases were solved and converted to CO_2_ production rates by the equation:

J CO_2_ = (12 * △CO_2_ * P * V) / (24 * R * (T_a_ + 273.15) (1)

where:

J CO_2_ = flux (µg CO_2_ h^-1^)

12 = µg C per µmole

△CO_2_ represents CO_2_ concentration increase (ppm/day)

P is the internal pressure (KPa)

V is the volume of the circulating gas (L), corrected for substrate volume included

24 is hours per day

R is the gas constant (8.314 L * kPa * ºK^-1^ mol^-1^)

T_s_ is the substrate temperature (ºC)

**CO_2_ isotopic analyses**. Periodically four times (*i.e.* linearly climbing period, the peak, after the peak and the end period) during the incubations based on pre-experiments, 4 small glass tubes each containing 2 ml of 2 M NaOH were placed into 3 replicate incubation bottles and those were capped to trap CO_2_ for 6 to 24 hours for ^13^C analysis. Strontium chloride (2 M) was then added into the NaOH solution to precipitate CO_2_. The resulting SrOH/SrCO_3_ precipitate was centrifuged 3 times at 1200 *g* for 10 min followed each time by rinsing with deionized, degassed water to remove SrOH until pH =7. The remaining solids were then dried at 105 °C and analyzed for ^13^C/^12^C ratios with an isotope-ratio mass spectrometer (MAT253, Finnigan MAT, Bremen, Germany). We interpolated those ^13^C values to solve the mixing model for other CO_2_ measurement times throughout the incubations. Previous studies (Ekblad and Högberg 2000, Samtruckova et al. 2000, Kelliher et al. 2005, Norris et al. 2013, Qiao et al. 2014) have shown that C isotope ratios vary smoothly during such incubations, so linear interpolations are appropriate.

**Substrate ^13^C isotopic ratios.** Samples of organic and mineral soils, leaf and wood litter were dried and ground to fine powder with a ball mill (MM200, Haan, Retsch, Germany). They were weighed into tin capsules to analyze for organic C and ^13^C/^12^C ratios by continuous-gas-flow isotope-ratio mass spectrometry (MAT253, Finnigan MAT, Germany), coupled with a ConFlo III device (Finnigan MAT, Germany) to an elemental analyzer (EA 1112, CE Instruments, Italy).

**Calculations and statistics.** Incubation data included respired CO_2_ flux rates and ^13^CO_2_ isotopic signatures. These were accumulated through time, and cumulative mean values and standard deviations were calculated. Standard deviations of cumulative CO_2_ fluxes, glucose release (%), priming and net C balances were calculated according to the following equations from Ku (1966).


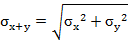
 (2)

where
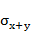
 is SD of the combined flux and
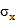
and
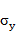
 are SDs of the individual fluxes.


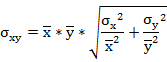
 (3)

Where
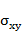
is SD of the combined flux,
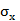
and
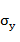
are SDs of the CO_2_ flux and SOC fractions, and
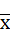
 and
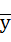
 are their respective mean values.

Flux-weighted δ^13^C and cumulative CO_2_ fluxes were input to the mixing model from Phillips & Gregg (2001) and Phillips *et al*. (2005), <http://www.epa.gov/wed/pages/models.htm>. The mixing model used to calculate fractions of CO_2_-C derived from organic matter (C_OC_) and from added glucose (C glucose). The two-end member mixing model was used for each measurement time, always starting from time zero because otherwise the OM substrate end member could not determined. One end member was the ^13^C-CO_2_ released from water-only treatments and the other was based on ^13^C-glucose added to the various substrates. This model allowed variability from mass-spectrometric measurements to be combined with that from CO_2_ flux measurements.

Based on the fractions of OM-derived CO_2_, primed C was calculated as follows:

Primed C = C_total_ – C_glucose_ – C_water only_ (4)

where C_total_ is total C-CO_2_ from glucose-treated OM substrates, C_glucose_ is C-CO_2_ derived from added glucose and C_water_ _only_ is total C-CO_2_ from the OM substrates receiving only water.

Shapiro-Wilkinson tests confirmed that all data were normally distributed. The relationships both additional amounts C and N for four OM forms priming effects were examined by two-way ANOVA followed by *post hoc* Tukey HSD tests (P<0.05; Tables S2 and S3).

**ESTIMATED GLOBAL PATTERNS OF DISSOLVED C AND N INPUTS**

Spatial patterns of C and N supply are distinct, with the former following biological productivity and the latter following regional N deposition.

Global N deposition has been mapped (Dentener et al. 2006), spanning 5 to 50 kg N ha^-1^ yr^-1^, but regional maps from Europe (Holland et al. 2005), North America (Holland et al. 2005, Zhang et al. 2012) and China (Ti et al. 2012) show large differences from that global pattern. Thus, more accurate N deposition maps should be developed. They might be further improved with fossil-fuel CO_2_ emission (Asefi-Najafabady et al. 2014) and global wildfire (Schulz et al. 2008) maps, if robust conversion factors for CO_2_ to NO_x_ emissions can be developed. This excludes anthropogenic biomass burning, which could make important regional contributions to the N cycle (Chen et al. 2010). Globally, maximum potential biological N fixation is 60 kg ha^-1^ yr^-1^ (Cleveland et al. 1999). Most important is that N emissions are regionally linked to N deposition because of rainout. We suggest that new dissolved-N inputs may range from 0.5 to 5 g m^-2^ yr^-1^ globally, but also that this map needs to be improved. We recognize that available N is generated by internal recycling within systems as well as from external inputs, but it should be higher with large external inputs (Matson et al. 2002).

In contrast, air concentrations of CO_2_ are globally mixed and do not follow regional emissions. Their effects on plant productivity and thence on labile C supply are controlled by climate (Field et al. 1998). High values of terrestrial NPP are about 1000 g C m^-2^ yr^-1^, while low values are around 100 g C m^-2^ yr^-1^ in deserts and very cold climates (Field et al. 1998). A high estimate of the NPP fraction becoming available C from canopy losses, root exudation and release of soluble C from decomposing litter is 10%, while low estimate of NPP becoming LOC is 2% (Helal and Sauerbeck 1984; Qualls et al. 1991; Hungate et al. 1997; Cheng and Johnson 1998; Moore and Dalva 2001; Michalzik and Stadler 2005; Phillips et al. 2008; Epron et al. 2011; Reinsch et al. 2013). While still poorly constrained, we suggest that global dissolved-C supply may range from 2 to 100 g C m^-2^ yr^-1^. The difficulty here is to link plant productivity to labile-carbon supply. Estimates exist for this, but there may be differences among vegetation types and other factors.

Global maps of labile C and N (and their ratios) can be developed, but with residual uncertainties. At local scales, uncertainties can be reduced by direct measurements of labile C and N concentrations. Extractions with water and dilute-salt solutions have been used for both C and N (Michalzik et al. 2001; Neff and Asner 2001; Ghani et al. 2003; Zsolnay 2003; McLauchlan and Hobbie 2004; Jones and Willett 2006; Guigue et al. 2014).

Under global change, more CO_2_ could increase NPP and thus root exudation and litter DOC on a global basis. Nitrogen inputs are expected to increase regionally with fuel combustion and agricultural fertilizer use. We stress that global C and N inputs already show large spatial variability and that such patterns will change in the future.

Here we applied C:N additions ranged from 1.25 to 320, generally following our presumption that global inputs may range from 0.4 to 200. We present evidence that decomposing substrates respond distinctly to labile C and N inputs. Our measured responses could form the basis for C and N resource driven OM decomposition models. Site-specific measures of available C and N are not challenging, but rarely have they been performed together. Our new perspective on priming with respect to C and N resources calls for direct testing elsewhere.

Table S1. Chemistry and C:N, C:P, N:P ratios in leaf and wood litter, soils from organic soil horizon and mineral soil A horizon from a subtropical forest at Ailao Mountain, Yunnan, China. Means ± standard errors from three replicates.

| Chemical species | Wood | Leaf | | Organic  Soil | | Mineral Soil | |
| --- | --- | --- | --- | --- | --- | --- | --- |
| Carbon (mg g^-1^) | 584.2±0.3 | 576.4±0.9 | | 424.5±0.4 | | 105.3±0.1 | |
| Nitrogen (mg g^-1^) | 3.05±0.01 | 14.12±0.03 | | 22.58±0.00 | | 6.48±0.01 | |
| Phosphorus (mg g^-1^) | 0.15±0.00 | 0.53±0.01 | | 1.28±0.01 | | 0.94±0.02 | |
| Available NH_4_^+^ (µg g^-1^) |  |  | | 25.1±1.2 | | 54±3.0 | |
| Available NO_3_^-^ (µg g^-1^) |  |  | | 15.8±0.4 | | 5.35±0.51 | |
| Available P (µg g^-1^) |  |  | | 5.81±0.14 | | 0.95±0.21 | |
| Available C (g kg-1) |  |  | | 16±0.7 | | 6.5±0.6 | |
| Substrate C:N | 192:1 | 41:1 | | 19:1 | | 16:1 | |
| Substrate C:P | 3895:1 | 1088:1 | | 332:1 | | 112:1 | |
| Substrate N:P | 20:1 | 27:1 | | 18:1 | | 7:1 | |
| Soil type | 1.00 | | 135.5 | | 8.50 | | 0.010 |

Table S2. Tukey HSD tests comparing differences in priming among substrates under the same treatment. The first label A, B or C indicates low, intermediate and high C additions and the second A, B or C the same for N additions. Different lower-case letters indicates significant differences between heterogeneous OMs at P<0.05.

| Treatments | OM substrates | | | |
| --- | --- | --- | --- | --- |
|  | Organic soil | Mineral soil | Leaf | Wood |
| AC | a | a | b | c |
| BC | a | a | b | c |
| CC | a | a | b | c |
| AB | a | a | a | b |
| BB | a | a | a | b |
| CB | a | b | c | d |
| AA | ac | b | c | d |
| BA | a | b | c | d |
| CA | a | b | c | c |

Table S3. Tukey HSD tests comparing differences in priming among three C addition levels or three N addition levels for the same heterogeneous OM. The first label A, B or C indicates low, intermediate and high C additions and the second A, B or C indicates the same for N additions. Different lower-case letters indicates significant difference between addition levels at P<0.05.

| OM substrates | Treatments | | |  | OM substrates | Treatments | | |
| --- | --- | --- | --- | --- | --- | --- | --- | --- |
|  | AA | AB | AC |  |  | AA | BA | CA |
| Organic soil | a | b | c |  | Organic soil | a | a | b |
| Mineral soil | a | b | c |  | Mineral soil | a | a | b |
| Leaf | a | b | c |  | Leaf | a | b | c |
| Wood | a | a | a |  | Wood | a | a | a |
|  |  |  |  |  |  |  |  |  |
|  | BA | BB | BC |  |  | AB | BB | CB |
| Organic soil | a | b | c |  | Organic soil | a | b | c |
| Mineral soil | a | b | c |  | Mineral soil | a | b | c |
| Leaf | a | b | c |  | Leaf | ab | b | c |
| Wood | a | b | c |  | Wood | a | a | b |
|  |  |  |  |  |  |  |  |  |
|  | CA | CB | CC |  |  | AC | BC | CC |
| Organic soil | a | b | c |  | Organic soil | a | a | b |
| Mineral soil | a | b | c |  | Mineral soil | a | b | c |
| Leaf | a | b | a |  | Leaf | a | a | a |
| Wood | a | b | a |  | Wood | a | a | b |

Table S4. Amounts and ratios of labile C and N added to four OM substrates in nine treatments. They are added on the basis of substrate organic C in each incubation. The first capital letters (*i.e.* A, B, and C, in black color) refer to the C addition levels, while the second capital letters (i.e. A, B and C, in red color) refer to the N addition levels. The numbers in brackets refer to C:N ratios after labile C and N was added for each treatment.

| C addition level | N addition level | | |
| --- | --- | --- | --- |
|  | A (0.015%OC) | B (0.06%OC) | C (0.24%OC) |
| A (0.3%OC) | AA (20:1) | AB (5:1) | AC (1.25:1) |
| B (1.2%OC) | BA (80:1) | BB (20:1) | BC(5:1) |
| C (4.8%OC) | CA (320:1) | CB (80:1) | CC (20:1) |

Table S5. Timing of CO_2_ flux-rate measurements during incubations for each substrate.

| Time (hours) | Leaf | Wood | | Organic  Soil | | Mineral  Soil | |
| --- | --- | --- | --- | --- | --- | --- | --- |
|  | 1.5  12  29  53  77  100  124  167  238  311  407  527  599  671  815  915 | 24  48  72  120  168  216  264  336  432  576  720 | | 16  22  48  117  165  240  288  336  358  384  428  452  474  500  524  575  630  676 | | 24  48  72  96  144  240  264  288  336  504  528 | |
| Soil type | 1.00 | | 135.5 | | 8.50 | | 0.010 |

Table S6. Results of two-way analysis of variance (ANOVA) to evaluate effects of C and N additions and interactions on cumulative priming effects. Bold numbers indicate significant differences at P < 0.05.

| Source of variation | df | MS | F | P-value |
| --- | --- | --- | --- | --- |
| Mineral soil |  |  |  |  |
| C addition | 2 | 181.55 | 9106.64 | **0.000** |
| N addition | 2 | 1.16 | 58.06 | **0.000** |
| C addition*N addition | 4 | 0.48 | 24.24 | **0.000** |
| Residuals | 63 | 0.02 |  |  |
| Soil type | 1.00 | 135.5 | 8.50 | 0.010 |
|  |  |  |  |  |
| Organic soil |  |  |  |  |
|  |  |  |  |  |
| C addition | 2 | 240.20 | 4075.9 | **0.000** |
| N addition | 2 | 82.83 | 1405.5 | **0.000** |
| C addition*N addition | 4 | 15.69 | 266.3 | **0.000** |
| Residuals | 45 | 0.06 |  |  |
|  |  |  |  |  |
| Leaf |  |  |  |  |
|  |  |  |  |  |
| C addition | 2 | 19.97 | 3.681 | **0.033** |
| N addition | 2 | 119.35 | 22.006 | **0.000** |
| C addition*N addition | 4 | 35.18 | 6.486 | **0.000** |
| Residuals | 45 | 5.42 |  |  |
| Wood  C addition  N addition  C addition*N addition  Residuals | 2  2  4  45 | 53.42  43.71  31.22  4.01 | 13.311  10.892  7.779 | **0.000**  **0.000**  **0.000** |
|  |  |  |  |  |

Table S7. Results of two-way analysis of variance (ANOVA) to evaluate effects of substrates and C:N ratios of added sources on cumulative priming effects. Bold numbers indicate significant differences at P < 0.05.

| Source of variation | df | MS | F | P-value |
| --- | --- | --- | --- | --- |
| Substrate | 3 | 459.0 | 91.303 | **0.000** |
| C:N ratios | 4 | 162.8 | 32.383 | **0.000** |
| Substrate * C:N ratios | 12 | 40.8 | 8.125 | **0.000** |
| Residuals | 196 | 5.0 |  |  |
| Soil type | 1.00 | 135.5 | 8.50 | 0.010 |
|  |  |  |  |  |

Figure S1 All results of priming for four organic matter forms during the incubation period. Values are means ± SD of 6-8 replicates. The first label A, B or C indicates low, intermediate and high C additions and the second A, B or C indicates low, intermediate and high N additions (Table S4).

**References cited in the supplemental material (in order of citation)**

Zhang, K. The characteristics of mountain climate in the north of Ailao Mts. Research of Forest Ecosystem on Ailao Mountains, Yunnan, (ed. Wu, Z.Y.), pp. 20–29. *Yunnan Science and Technology Press,* Kunming, China (1983).

WRB, World Reference Base for Soil Resources. *World Soil Resources Reports* 103, FAO, Rome (2006).

Wu, Z. Y. Vegetation of China. *Science Press*, Beijing (1980).

Chan, O. C. *et al.* 16S rRNA gene analyses of bacterial community structures in the soils of evergreen broad-leaved forests in south-west China. *FEMS Microbiol. Ecol.* **58,** 247–259 (2006).

Feng, W., Schaefer, D.A., Zou, X., Zhang, M. Shifting sources of soil labile organic carbon after termination of plant carbon inputs in a subtropical moist forest of southwest China. *Ecol. Res.* **26,** 437–444 (2011).

Burns, R. G. *et al.* Soil enzymes in a changing environment: Current knowledge and future directions. *Soil Biol. Biochem.* **58,** 216-234 (2013).

Manzoni, S. & Porporato, A. Common hydrologic and biogeochemical controls along the soil–stream continuum. *Hydrol. Proc.* **25,** 1355–1360 (2011).

Güsewell, S. & Gessner, M. O. N:P ratios influence litter decomposition and colonization by fungi and bacteria in microcosms. *Funct. Ecol.* **23,** 211–219 (2009).

Cleveland, C. C., Townsend, A. R. & Schmidt, S. K. Phosphorus limitation of microbial processes in moist tropical forests: evidence from short-term laboratory incubations and field studies. *Ecosystems* **5,** 680-691 (2002).

Norris, C. E., Quideau, S. A. & Macey, D. E. Processing of ^13^C glucose in mineral soil from aspen, spruce and novel ecosystems in the Athabasca oil sands region. *Appl. Soil Ecol.* **71,** 24-32 (2013).

Qiao, N. *et al*. Labile carbon retention in forest soils compensates for CO_2_ released by priming. *Glob. Change Biol.* **20,** 1943-1954 (2014).

Ku, H. Notes on the use of propagation of error formulas. *J. Res. Nat. Bur Stand C Engineer Instr.* **70,** 263–273 (1966).

Phillips, D. L. & Gregg, J. W. Uncertainty in source partitioning using stable isotopes. *Oecologia* **127,** 171–179 (2001).

Phillips, D. L., Newsome, S. D. & Gregg, J. W. Combining sources in stable isotope mixing models: alternative methods. *Oecologia* **144,** 520–527 (2005).

Dentener, F *et al.* Nitrogen and sulfur deposition on regional and global scales: A multimodel evaluation. *Glob. Biogeochem. Cyc.* **20,** GB4003. DOI: 10.1029/2005GB002672 (2006).

Holland, E. A., Braswell, B. H., Sulzman, J. & Lamarque, J-F. Nitrogen deposition onto the United States and Western Europe: synthesis of observations and models. *Ecol. Appl.* **15(1),** 38-57 (2005).

Zhang, L. *et al.* Nitrogen deposition to the United States: distribution, sources, and processes. *Atmos. Chem. Phys. Discuss.* **12,** 241–282 (2012).

Ti, C., Pan, J., Xia, Y. & Yan, X. A nitrogen budget of mainland China with spatial and temporal variation. *Biogeochemistry* **108,** 381–394 (2012).

Asefi-Najafabady, S. P. J. *et al.* A multiyear, global gridded fossil fuel CO_2_ emission data product: Evaluation and analysis of results. *J. Geophys. Res. Atmos.* **119,** 10213–10231 (2014).

Schultz, M. G. *et al*. Global wildland fire emissions from 1960 to 2000. *Glob Biogeochem Cyc* **22,** GB2002 (2008).

Chen, Y. *et al.* Nitrogen deposition in tropical forests from savanna and deforestation fires. *Glob. Change Biol.*[**16(7**](http://onlinelibrary.wiley.com/doi/10.1111/gcb.2010.16.issue-7/issuetoc)**),** 2024–2038 (2010).

Cleveland, C. C. *et al*. Global patterns of terrestrial biological nitrogen (N_2_) fixation in natural ecosystems. *Global Biogeochem. Cyc.* **13,** 623-645 (1999).

Matson, P. A., Lohse, K. A. & Hall, S. J. The globalization of nitrogen deposition: consequences for terrestrial ecosystems. *Ambio* **31(2),** 113-119 (2002).

Field, C. B., Behrenfeld, M. J., Randerson, J. T. & Falkowski, P. Primary production of the biosphere: integrating terrestrial and oceanic components. *Science* **281,** 237-240 (1998).

Helal, H. M. & Sauerbeck, D. R. Influence of plant roots on C and P metabolism in soils. *Plant Soil* **76,** 175-182 (1984).

Qualls, R. G., Haines, B. L. & Swank, W. T. Fluxes of dissolved organic nutrients and humic substances in a deciduous forest. *Ecology* **72,** 254-266 (1991).

Hungate, B. A. *et al.* The fate of carbon in grasslands under carbon dioxide enrichment. *Nature* **388,** 576-579 (1997).

Cheng, W. X. & Johnson, D. W. Elevated CO_2_, rhizosphere processes, and soil organic matter decomposition. *Plant Soil* **202,** 167–174 (1998).

Moore, T. R. & Dalva, M. Some controls on the release of dissolved organic carbon by plant tissues and soils. *Soil Sci.* **166,** 38–47 (2001).

Michalzik, B. & Stadler, B. Importance of canopy herbivores to dissolved and particulate organic matter fluxes to the forest floor. *Geoderma* **127(3-4),** 227-236 (2005).

Phillips, R. P., Erlitz, Y., Bier, R. & Bernhardt, E. S. New approach for capturing soluble root exudates in forest soils. *Funct Ecol.* **22,** 990–999 (2008).

Epron, D. *et al.* Seasonal variations of belowground carbon transfer assessed by *in situ* ^13^CO_2_ pulse labelling of trees. *Biogeosciences* **8,** 1153–1168 (2011).

Reinsch, S. *et al*. Short-term utilization of carbon by the soil microbial community under future climatic conditions in a temperate heathland. *Soil Biol. Biochem.* **68,** 9-19 (2013).

Michalzik, B., Kalbitz, K., Park, J. H., Solinger, S. & Matzner, E. Fluxes and concentrations of dissolved organic carbon and nitrogen—a synthesis for temperate forests. *Biogeochemistry* **52,** 173–205 (2001).

Neff, J. C. & Asner, G. P. Dissolved organic carbon in terrestrial ecosystems: synthesis and a model. *Ecosystems* **4,** 29–48 (2001).

Ghani, A., Dexter, M. & Perrott, K. W. Hot-water extractable carbon in soils: a sensitive measurement for determining impacts of fertilisation, grazing and cultivation. *Soil Biol. Biochem.* **35,** 1231–1243 (2003).

Zsolnay, A. Dissolved organic matter: artefacts, definitions, and functions. *Geoderma* **113,** 187–209 (2003).

McLauchlan, K. K. & Hobbie, S. E. Comparison of labile soil organic matter fractionation techniques. *Soil Sci. Soc. Am. J.* **68,** 1616–1625 (2004).

Jones, D. L. & Willett, D. B. Experimental evaluation of methods to quantify dissolved organic nitrogen (DON) and dissolved organic carbon (DOC) in soil. *Soil Biol, Bi*o*chem*.[**38(5**](http://www.sciencedirect.com/science/journal/00380717/38/5)**),** 991–999 (2006).

Guigue, J. *et al.* A comparison of extraction procedures for water-extractable organic matter in soils. *Eur J. Soil Sci.* **65,** 520–530 (2014).
